# nf-core/pacvar: a pipeline for analyzing long-read PacBio whole genome and repeat expansion sequencing data

**DOI:** 10.1093/bioinformatics/btaf116

**Published:** 2025-03-17

**Authors:** Tanya Jain, Claire Clelland

**Affiliations:** Weill Institute for Neurosciences, University of California, San Francisco, CA, 94158, United States; Weill Institute for Neurosciences, University of California, San Francisco, CA, 94158, United States; Department of Neurology, Memory & Aging Center, University of California, San Francisco, CA, 94158, United States

## Abstract

**Motivation:**

Pacific Biosciences (PacBio) single-molecule, long-read sequencing enables whole genome annotation and the characterization of 20 complex repetitive repeat regions, especially relevant to neurodegenerative diseases, through their PureTarget panel. Long-read whole-genome sequencing (WGS) also allows for the detection of structural variants that would be difficult to detect with traditional short-read sequencing. However, the raw unaligned Binary Alignment Map data need to be processed before analysis. There is a need for an intuitive comprehensive bioinformatic pipeline that can analyze these data.

**Results:**

We present nf-core/pacvar, a comprehensive pipeline for analyzing both PacBio single-molecule PureTarget and WGS data that demultiplexes and parallelizes pre-processing, variant calling and repeat characterization. nf-core/pacvar is compatible with little configuration and has few dependencies. This pipeline enables rapid end-to-end, parallel processing of PacBio single-molecule whole genome and targeted repeat expansion sequencing.

**Availability and implementation:**

nf-core/pacvar is available on nf-core website (https://nf-co.re/pacvar/) and on github (https://github.com/nf-core/pacvar) under MIT License (DOI: 10.5281/zenodo.14813048).

## 1 Introduction

Long-read sequencing has made characterizing complex genomic regions possible, including centromeric regions, repeat regions, and those regions with high G/C+ content possible. Many diseases are linked to repeat expansions, such as C9orf72-related frontotemporal dementia, amyotrophic lateral sclerosis, spinocerebellar ataxia, and Friedreich ataxia. Long-read sequencing is especially promising for understanding repeat-expansion diseases, as the entire repeat expansion can be accurately characterized by sequencing single molecules of DNA, thereby informing the biological investigation of the repeat expansion, potentially increasing diagnostic accuracy, and informing the effectiveness of gene-targeting therapeutic strategies (Salomonsson *et al.* 2024, Tsai *et al.* 2024). Pacific Biosciences (PacBio) technology circulates DNA through the addition of SMRTbell adapters. The resulting circularized DNA can be sequenced continuously in multiple passes, creating subreads that are then used to produce a highly accurate long consensus read (Wenger *et al.* 2019). In addition to whole-genome sequencing (WGS), PacBio’s PureTarget panel allows for targeted sequencing of repeat expansion loci relevant to neurodegenerative diseases, additionally capturing methylation data, precise sizing of the repeat expansions and mosaicism, and creating a trove of clinically and scientifically informative data.

There is a need for an intuitive comprehensive bioinformatic pipeline that can analyze PacBio PureTarget WGS long-read data. Our pipeline expands upon the functionality of existing toolkits such as SMRTlink (https://www.pacb.com/smrt-link/) and PacBio’s latest released pipeline, named HiFi-human-WGS-WDL (https://github.com/PacificBiosciences/HiFi-human-assembly-WDL), by enabling a single-command execution and having compatibility with multiple backends. The HiFi-human-WGS-WDL pipeline, when run with the miniwdl execution engine, is currently not compatible with High-Performance Computing (HPC) clusters that do not have a SLURM scheduler, nor with Google Cloud Platform. The SMRTlink toolset cannot be installed on MacOS operating systems and necessitates singularity to run variant calling. Further, the setup and optimization of a workflow manager can be a non-trivial task. Therefore, in addition to its compatibility across infrastructures and its ability to run with multiple containerization methods, nf-core/pacvar provides researchers with choice in being implemented with the alternative workflow manager—Nextflow. nf-core/pacvar also expands on existing toolkits by not requiring researchers to apply each intermediate processing tool to their samples individually. While there is a Nextflow pipeline available for RNA long-read sequencing data (Guizard *et al.* 2023), nf-core/isoseq, one does not yet exist for long-read DNA data. Pre-processing PacBio data requires demultiplexing samples before aligning PacBio’s un-aligned Binary Alignment Map (BAM) files, followed by sorting and indexing steps. For WGS, it is then necessary to variant call single-nucleotide variants and structural variants before phasing and then again sort and index the resulting variant call files. For PureTarget analysis, post-processing requires additional genotyping of repeat regions in the repeat expansion—assessing the repeat number, mosaicism, and variants in the motifs—before visualization. Here, we submit the nf-core/pacvar workflow to integrate tools specifically designed for PacBio data as well as other bioinformatics packages. Lima is used for demultiplexing, pbmm2 for alignment, and SAMtools (Li *et al.* 2009) indexing and sorting BAMs. Deepvariant (Poplin *et al.* 2018), HaplotypeCaller (Poplin *et al.* 2017), and pbsv are used for variant calling. HiPhase (Holt *et al.* 2024) is used for phasing, as well as BCFtools (Danecek *et al.* 2021) and Tabix (Li 2011) for VCF processing. For repeat expansion analysis, the workflow uses TRGT (Dolzhenko *et al.* 2024) for repeat genotyping and visualization; BCFtools and SAMtools are used for sorting and indexing. nf-core/pacvar is implemented in Nextflow (Di Tommaso *et al.* 2017) and hosted as an nf-core pipeline, an open-source repository where it is actively maintained and adheres to strict testing guidelines ensuring accessibility and reproducibility. These strict guidelines include hosting test-datasets in which continuous integration tests are run with whenever there is a pipeline update. nf-core also requires the pipeline have usage examples in the form of executable commands, a description of all generated result files, and rigorous documentation. Further, as being part of the nf-core ecosystem, the pipeline benefits from the domain expertise of maintainers and the nf-core community, who are accessible for questions through the nf-core slack. Comprehensively, analyzing PacBio long-read DNA would require running all programs for each sample after demultiplexing, which is tedious and error-prone. The workflow presented here allows for parallel processing to increase throughput and accuracy and has the added flexibility of allowing users to post process the more specific PureTarget panel. nf-core pacvar therefore enables precise, accurate, tailored, and efficient long-read sequencing analysis with minimal setup. Our pipeline makes PacBio long-read experiments accessible to researchers with varied skillsets.

## 2 Pipeline description and implementation

### 2.1 Implementation

**Figure 1.**
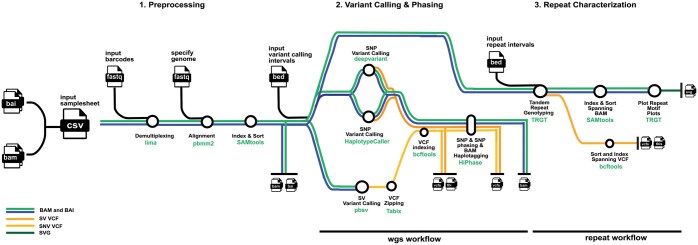
The Pacvar pipeline comprised of (1) Pre-processing, (2) Variant Calling and Phasing, and (3) Repeat Characterization. The component software packages are noted at each step of the pipeline as well as the associated function.

Broadly, the pipeline has two main workflows: (i) the wgs workflow and (ii) the repeat workflow. The pipeline is comprised of three main components: (i) Pre-processing, (ii) Variant Calling and Phasing, and (iii) Repeat Characterization as displayed in Figure 1. The nf-core/pacvar pipeline is written in Nextflow and follows the nf-core template, which adheres to nf-cores standardized best-practice guidelines (Ewels *et al.* 2020). All software the pipeline uses are available as Docker, Singularity containers, Apptainer, through biocontainers or conda (Da Veiga Leprevost *et al.* 2017; Kurtzer *et al.* 2017; Grüning *et al.* 2018), which allow each component of the pipeline to be reproducible on a on various infrastructures such as HPC systems with different schedulers, on local systems, as well as cloud providers. The modularization of the pipeline through nf-core’s module template also allows each module in the pipeline to be easily and individually updated when there are package changes. The requirements to run the pipeline are infrastructure with a unix-based operating system, an installation of Java 17 or later, download of the Nextflow executable, and at least one containerization solution.

### 2.2 Pre-processing

PacBio supports multiplexing samples. Their library prep protocols involve ligating barcodes to individual samples (Tsai *et al.* 2024). Multiple samples can then be run on the same flow cell on the Revio sequencing machine, which produces an unaligned BAM output containing the reads from the multiplexed samples in a compressed binary form. These BAM files need to be demultiplexed before being individually processed. In nf-core/pacvar, each run output can be optionally demultiplexed with lima. While demultiplexing can be performed on the Revio and Vega instrument, the pipeline’s demultiplexing feature enables after the fact demultiplexing even when the setting is not applied during sequencing. Lima also offers customizable parameters such as min-length, which omits reads below a specified length after demultiplexing and can be specified as a parameter in custom config files. The inclusion of lima in nf-core/pacvar, therefore, allows a user to tailor the processing according to their use case. Then each demultiplexed sample is aligned to the specified reference genome with pbmm2 to generate aligned BAM files. These BAM files are subsequently sorted with and indexed with SAMtools so they can be used in standard visualization tools such as Integrative Genomics Viewer (Thorvaldsdóttir *et al.* 2013).

### 2.3 Variant calling and phasing

nf-core/pacvar allows users to choose between Google’s deepvariant (Poplin *et al.* 2018) and the Broad Institute’s HaplotypeCaller (Poplin *et al.* 2017) for single-nucleotide polymorphism variant calling. Google’s deepvariant uses a convolutional neural network to classify each locus as genetic variation or sequencing error. Alternatively, HaplotypeCaller establishes structural variants based on how much variation the loci have relative to the reference. Lin *et al.* (2022) found that deepvariant had shorter execution time and higher accuracy as compared to HaplotypeCaller on clinical samples. However, deepvariant has no current support for conda. To allow usage across platform configurations, nf-core/pacvar gives the user the optionality to specify either. Additionally, by supporting both variant callers, the pipeline facilitates downstream analysis to assess the concordance of the two single-nucleotide variant callers on samples.

Long-read sequencing is uniquely poised to assess structural variations due to its ability to span larger genomic regions without breaks. nf-core/pacvar uses pbsv to call structural variants. The single-nucleotide and structural variants are then phased with HiPhase, assigning each variant to a specific allele. We have found this feature to be important for understanding both the impact of methylation and the outcome of CRISPR edits in an allele-specific manner (Salomonsson *et al.* 2024). Further, the pipeline can skip calling structural variants, single-nucleotide variants, or phasing to enable the pipeline to be tailored to individual use cases.

### 2.4 Repeat expansion characterization

PacBio’s PureTarget panel offers the characterization of 20 genes associated with neurological disorders: *ATN1, ATXN1, ATXN2, ATXN3, ATXN7, ATXN8, ATXN10, CACNA1A, PPP2R2B, TBP, FXN, RFC1, FMR1, HTT, DMPK, CNBP, C9orf72, TCF4, AR,* and *PABPN*. The target regions are excised from high-molecular-weight DNA, adapters are added to cut ends, and up to 48 samples are multiplexed and sequenced on PacBio’s Revio or Vega. The produced PureTarget reads are genotyped with TRGT to produce BAM and VCF files that span the targeted repetitive regions. These BAM and VCF files are sorted and indexed with SAMtools and BCFtools, respectively. Next nf-core/pacvar uses TRGT to visualize the repetitive regions and displays waterfall plots that allow for visualization of the mosaicism and repeat motifs on each allele. nf-core/pacvar will also work for other repeat expansion loci that have undergone targeted enrichment. In this scenario, the main change is the library prep, which would need to be customized to enrich the new expansion repeat loci. The BED file containing the intervals to plot should also be modified to the coordinates of the new repeat region.

### 2.5 Testing and output reporting

The pipeline is available at https://nf-co.re/pacvar/ and has been validated with unit tests in accordance with nf-core standards. Users can test the pipeline with hosted test datasets and test configuration files for both the repeat workflow and wgs workflow, which produce outputs as detailed in https://nf-co.re/pacvar/dev/docs/output/. Additionally, the pipeline generates a MultiQC report that summarizes the execution runtime and resource usage, as well as confirms the pipeline’s successful execution (Ewels *et al.* 2016). When running the pipeline with test profiles, users can examine the MultiQC report to assess performance metrics such as execution times and memory usage, helping them gauge the pipeline’s behavior on their own infrastructure. This report confirms the runs successful execution and provides insights into its computational requirements. Allocated CPU and memory can be changed as needed through a custom configuration file, as detailed in https://nf-co.re/docs/usage/getting_started/configuration.

## 3 Conclusion

nf-core/pacvar is a scalable, easily deployable, and efficient pipeline for analyzing PacBio long-read DNA sequencing. It is also compatible with different HPC schedulers, cloud environments, and local systems. The pipeline has the flexibility to analyze both PacBio PureTarget and WGS data with the ability to skip certain user-specified steps, making the pipeline versatile and comprehensive.

## Data Availability

nf-core/pacvar is available on nf-core website (https://nf-co.re/pacvar/) and on github (https://github.com/nf-core/pacvar) under MIT License (DOI 10.5281/zenodo.14813048).
